# The Story of the Insane from Year to Year

**Published:** 1905-01-07

**Authors:** 


### THE STORY OF THE INSANE FROM YEAR TO YEAR.

Seventeenth Annual Series.

### GLAMORGAN COUNTY ASYLUM AT BRIDGEND.

On the first of January this asylum contained 1,910 patients, and the number had risen to 1,933 at the end of the year. There were 447 first admissions, 96 readmissions, 138 recoveries, 41 discharged relieved, 110 discharged not improved, and 231 died. The average number resident was 1,905, and the total number under treatment was 2,441. The recoveries stand at 27·7 per cent. of the admissions, and the deaths at 9·4 per cent. of the average number resident. Of the year's deaths 78 were caused by cerebral and spinal diseases (general paralysis alone accounting for 48 of these), 36 by consumption, 11 by other lung diseases, and 1 by colitis. Of the year's admissions 97 were supposed to owe their insanity to such moral causes as domestic trouble, adverse circumstances, and mental anxiety; while among the physical causes we have hereditary influence heading the list with 161, and intemperance in drink following with 141. The asylum has been somewhat crowded, but with the removal of the cases chargeable to the Borough of Cardiff this over-crowding would cease. Dr. Pringle shows that Glamorganshire has only one lunatic to every 499 persons, whereas the Borough of Swansea has one to every 310, and Cardiff one to every 265; and he thinks the county owes its mental healthiness to the fact of its exceedingly mixed population, one-third consisting of people not born within its borders. The great event of the year in the Glamorgan Asylum was the resignation of Dr. Pringle. The Committee voted him a pension of £1,000 which we hope he may long enjoy. Dr. Stewart, formerly senior assistant medical officer, was appointed his successor. The weekly maintenance rate is 9s. 4d. per head.

### PORTSMOUTH BOROUGH ASYLUM.

The number on the first of January was 679, and on December 31st it was 699. There were 176 first admissions, 34 readmissions, 97 recoveries, 10 discharged relieved, and 80 deaths. The average number resident was 693, and the total number under care during the year was 889. The recoveries were 47·13 per cent. of the admissions, and the deaths were 11·5 per cent. of the average number resident. Both of these percentages are high, the recoveries decidedly so. Of the year's deaths 24 were caused by cerebral and spinal diseases, 16 by consumption, 1 by pneumonia, 1 by enteric fever, and 15 by colitis. Of the latter disease there were 52 cases, and, what is comparatively rare, this number included three nurses. Dr. Mumby does not give us his views of the origin of the disease, nor of its propagation; and although there were 16 deaths from phthisis it does not appear whether any steps are being taken to isolate the cases suffering from this disease or whether the open-air treatment has been adopted for them. Beer has been discontinued as an article of diet, and Dr. Mumby remarks that "those patients whose excessive indulgence in alcoholic liquors was responsible for bringing them to this institution, no longer have the temptation daily brought before them, and can learn from their own observation that alcohol is not necessary as an article of food." At all events there can be no doubt that the money might be much better spent than on beer, and we hope that some harmless luxury will be provided in place of it. The asylum is nearly full, but there are 156 Southampton cases and 24 from other parishes. There are also 50 private patients who pay from 14s. to 42s. a week. The latter sum is much too high for a public asylum to charge as it brings rate-supported institutions into competition with private asylums. The weekly charge for paupers is 10s. 10d. per head per week. The Committee say that the asylum "has been managed favourably during the past twelve months." Dr. Mumby has two assistant medical officers, and, very properly, one of these is a lady. The report of the Commissioners in Lunacy is not given.

